# Thermodynamic, Spatial and Methodological Considerations for the Manufacturing of Therapeutic Polymer Nanoparticles

**DOI:** 10.1007/s11095-020-2783-4

**Published:** 2020-02-24

**Authors:** Sara Maslanka Figueroa, Daniel Fleischmann, Sebastian Beck, Achim Goepferich

**Affiliations:** Department of Pharmaceutical Technology, University of Regensburg, Universitaetsstrasse 31, 93053 Regensburg, Germany

**Keywords:** encapsulation, microfluidics, nanoprecipitation, pirfenidone, polymeric nanoparticles

## Abstract

**Purpose:**

Evaluate fundamental parameters that dictate the effectiveness of drug loading.

**Methods:**

A model water-soluble drug lacking ionizable groups, pirfenidone (PFD), was encapsulated through nanoprecipitation in poly(ethylene glycol)-poly(lactic acid) (PEG-PLA)-poly(lactic-co-glycolic acid) (PLGA) NPs. Firstly, the thermodynamic parameters predicting drug-polymer miscibility were determined to assess the system’s suitability. Then, the encapsulation was evaluated experimentally by two different techniques, bulk and microfluidic (MF) nanoprecipitation. Additionally, the number of molecules that fit in a particle core were calculated and the loading determined experimentally for different core sizes. Lastly, the effect of co-encapsulation of α-lipoic acid (LA), a drug with complementary therapeutic effects and enhanced lipophilicity, was evaluated.

**Results:**

The thermodynamic miscibility parameters predicted a good suitability of the selected system. MF manufacturing enhanced the encapsulation efficiency by 60–90% and achieved a 2-fold higher NP cellular uptake. Considering spatial constrictions for drug encapsulation and increasing the size of the PLGA core the number of PFD molecules per NP was raised from under 500 to up to 2000. More so, the co-encapsulation of LA increased the number of drug molecules per particle by 96%, with no interference with the release profile.

**Conclusions:**

Thermodynamic, spatial and methodological parameters should be considered to optimize drug encapsulation.

**Electronic supplementary material:**

The online version of this article (10.1007/s11095-020-2783-4) contains supplementary material, which is available to authorized users.

## Introduction

Nanoprecipitation (1), or solvent displacement, is a frequently used technique for the preparation of therapeutic polymer nanoparticles (NPs), as it is a simple, clean, and versatile approach (2). More so, it enables drug encapsulation without requiring additional steps, such as covalently coupling the drug to structural particle components.

There are several elements that to some extent determine the success of a nanoprecipitation-mediated drug encapsulation, such as the physicochemical characteristics of the selected drug. As this technique involves the addition of a small volume of organic polymer phase into a large volume of aqueous phase, it has mostly been exploited for the encapsulation of lipophilic drugs that have little to no water solubility. Its application to water-soluble drugs is often inefficient, but can be improved to some extent by modulating the pH value of the water phase (3–5) or promoting electrostatic interaction with excipients (4). Alternatively, some authors have resulted to modify the drug molecule itself (6), or released it from its salt form (2), for which doxorubicin is a common example (7). However, these approaches are not universally applicable. For the former the presence of ionizable groups is a mandatory requirement and the latter may not be a feasible option due to the additional regulatory burdens associated with changing a drug’s structure. More so, there is a lack of research regarding the encapsulation through nanoprecipitation of water-soluble molecules that lack the aforementioned criteria.

An additional element that has an impact on drug encapsulation is the thermodynamic compatibility, i.e. miscibility, of the drug and particle-forming polymers. Therefore, thermodynamic parameters such as the Flory-Huggins interaction parameter (χ_sp_), or the solubility parameters (δ) are frequently used to assess drug-polymer miscibility (8). However, there are other elements that can determine the successful encapsulation of a drug in a selected particle system, such as dimensional restrictions, the used nanoprecipitation method or the presence of a co-encapsulated drug molecule.

Therefore, the goal of this study was to encapsulate through nanoprecipitation a partially water-soluble drug lacking ionizable groups and evaluate different parameters dictating the drug loading.

**Fig. 1:**
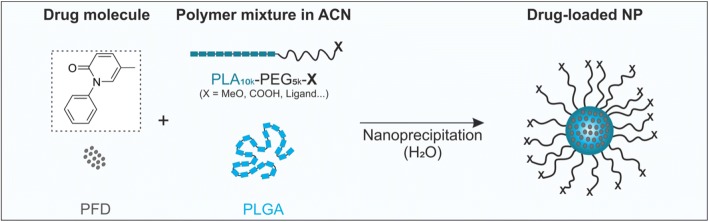
Encapsulation of PFD in block copolymer NPs. Particles are prepared through nanoprecipitation of organic mixtures of PEG-PLA, PLGA and the drug in aqueous medium.

As a drug model for our purposes we selected pirfenidone (PFD) (Fig. 1). PFD is a small molecule drug, with a log *P* = 2.14. Therefore, it would be initially considered a good candidate for encapsulation through nanoprecipitation. However, it is soluble in water up to 2 mg/mL, which is exceedingly high for this technique, due to the large volumes of water phase being used.

PFD is an antifibrotic agent, approved for the treatment of idiopathic pulmonary fibrosis (9). Additionally, recent studies showed its potential for the treatment of diabetic kidney disease (10,11) and glomerulosclerosis (12). However, it has a considerable plasma protein binding (50%) and fast elimination half-life (2.4 h), which requires a large oral dose intake (> 2 g/day) to achieve therapeutic effects (13). This elevated daily intake prompts considerable gastrointestinal side effects which contribute to therapy discontinuation by a large number of patients (11,14). Therefore, it is an excellent candidate that would benefit from incorporation in a nanoparticulate system. Over the past years PFD has been encapsulated in PLGA NPs by the emulsion solvent evaporation method for the treatment of pulmonary fibrosis (15) and corneal wounds (16), in chitosan-alginate nanocarriers through the pre-gelation method for transdermal delivery (17) and in liquid crystalline nanoparticles (18). However, it has never been encapsulated before by means of nanoprecipitation, which would considerably ease NP preparation and allow for simple cost-effective upscaling and reproducible results (19).

For our systematic investigation of the PFD encapsulation we relied on well-established poly(ethylene glycol)-poly(lactic acid) (PEG-PLA) block copolymer NPs (20) with a poly(lactic-coglycolic acid) (PLGA)-stabilized core (Fig. 1). Such particles are known for their good biocompatibility and highly tunable composition. Additionally, by modifying the PEG end-groups with ligands the particles can be targeted to receptors on the cellular membrane to increase their specificity (21). To evaluate the suitability of our particle system for the encapsulation of PFD we firstly estimated the compatibility of PFD with the particle-forming polymers through the calculation of thermodynamic miscibility parameters. Then, we prepared the NPs through two different nanoprecipitation techniques, bulk and microfluidic (MF) manufacturing, and assessed the drug loading. More so, we investigated the influence of the two different approaches on the NP characteristics and their interaction at cellular level. Additionally, we evaluated the spatial constrain on drug loading by calculating the number of molecules that can fit in a single NP core and experimentally determined the influence of this parameter by preparing particles with larger cores. Finally, we investigated the effect of introducing an additional drug molecule in the formulation, α-lipoic acid (LA), on PFD encapsulation.

## Materials and Methods

### Materials

Methoxy and carboxylic acid end-functionalized PEG (MeO-PEG_5k_-OH and COOH-PEG_5k_-OH) with a molecular weight of 5000 Da were purchased from JenKem Technology USA Inc. (Allen, TX, USA). PFD was obtained from MedChem Express (Sollentuna, Sweden). Dulbecco’s phosphate-buffered saline (DPBS) was purchased from Thermo Fisher Scientific (Waltham, MA, USA). LA, PLGA with a molecular weight of 13.4 kDa, and all other reagents and chemicals in analytical grade were purchased from Sigma Aldrich (Taufkirchen, Germany). Ultrapure water for particle preparation was obtained from a Millipore Milli-Q water purification system (Billerica, MA, USA).

### Compatibility of Drug and Particle-Assembling Polymers

To predict the miscibility of particle-forming polymers with either PFD or LA, different thermodynamic parameters were calculated. To that end, an ideal particle polymer distribution was assumed where PEG conforms the shell, PLGA the core, and PLA is situated at the core-shell interphase. The interference of concurring NP polymers in the miscibility was not taken into consideration. The total solubility parameters (δ) were obtained from literature values for the polymers (PEG (24.0 MPa^1/2^) (22), PLA (22.0 MPa^1/2^) (22) and PLGA (22.3 MPa^1/2^) (23)), and calculated for PFD and LA using the partial solubility parameters after Krevelen and Nijenhuis (24) determined by the group contributions methods using Eq. 1–3. δ_d,_ δ_p_, δ_h_ are the partial solubility parameters associated with the dispersion forces, the polar interactions and the hydrogen bonding components, respectively.

1 $$ {\updelta}_d=\sum \frac{F_{di}}{V} $$

2 $$ {\updelta}_p=\frac{{\left(\sum {F}_{pi}^2\right)}^{\raisebox{1ex}{$1$}\!\left/ \!\raisebox{-1ex}{$2$}\right.}}{V} $$

3 $$ {\updelta}_h={\left(\sum \frac{E_{hi}}{V}\ \right)}^{\raisebox{1ex}{$1$}\!\left/ \!\raisebox{-1ex}{$2$}\right.} $$

The estimation of the molecular volume of PFD (*V =* 119 cm^3^) and LA (*V =* 144 cm^3^) was done by the group contribution methods after Fedors (25). The total solubility parameters were determined using Eq. 4 (24).

4 $$ {\delta}^2={\updelta}_d^2+{\updelta}_p^2+{\updelta}_h^2 $$

The differences in solubility parameters (Δδ, Δδ_d,_ Δδ_p_, Δδ_h_) were calculated by subtracting the polymer’s from the drug’s solubility parameter. The mixing enthalpy calculated from the total- or partial solubility parameters (ΔH_MT_ and ΔH_M_, respectively) was determined according to Eq. 5 (26) and 6 (27), where Φ_1_ and Φ_2_ and δ_1_ and δ_2_ represent the volume fractions and the solubility parameters of the drug and polymer, respectively.

5 $$ \Delta  {H}_{MT}={\phi}_1{\phi}_2{\left({\delta}_{drug}-{\delta}_{polymer}\right)}^2 $$

6 $$ \Delta  {H}_M={\phi}_1{\phi}_2\left[{\left({\delta}_{d1}-{\delta}_{d2}\right)}^2+{\left({\delta}_{p1}-{\delta}_{p2}\right)}^2\right.+\left.{\left({\delta}_{h1}-{\delta}_{h2}\right)}^2\right] $$

The Flory-Huggins interaction parameter (χ_sp_) was calculated with the Hildebrand-Scatchard equation (Eq. 7) using the obtained total solubility parameters.

7 $$ {\chi}_{sp}={\left({\delta}_1-{\delta}_2\right)}^2\frac{V_{drug}}{RT} $$

### Polymer Synthesis

Block copolymers (PEG_5k_-PLA_10k_) with different PEG-end functionalization (MeO, or COOH) were synthesized after Qian *et al.* (28) with slight modifications as previously described by our group (29). In brief, for the ring opening polymerization of cyclic 3,6-dimethyl-1,4-dioxane-2,5-dione (lactide), MeO-PEG_5k_-OH and COOH-PEG_5k_-OH were used as macroinitiators using 1,8-diazabicyclo[5.4.0]undec-7-ene (DBU) as a catalyst. As products, MeO-PEG_5k_-PLA_10_ and COOH-PEG_5k_-PLA_10k_ were obtained, for non-targeted and targeted NP preparation, respectively (please refer to supplementary methods for ligand modification of the polymers).

### Particle Preparation Via Bulk or Microfluidic Nanoprecipitation

Block copolymer NPs were prepared through nanoprecipitation of PEG_5k_-PLA_10k_ and PLGA_13.4k_ polymer mixtures. To that end, and if not indicated otherwise, PEG-PLA and PLGA were mixed at a 70:30 mass ratio in acetonitrile (ACN) to a final polymer concentration of 10 mg/mL (1 mL). For drug-loaded particles, different amounts of PFD ranging from 25 μg to 10 mg (1–600-fold molar excess to PLGA) were added to the polymer mixture. Afterwards, for bulk nanoprecipitation, they were pipetted in stirring sterile filtrated Millipore water (5 mL) at a 1:5 organic to aqueous phase ratio to a polymer concentration of 2 mg/mL.

For MF manufacturing, the particles were prepared using the NanoAssemblr™ Benchtop (Precision NanoSystems Inc., Vancouver, Canada). Process parameters were controlled using the NanoAssemblr™ controller software (v1.09). If not noted otherwise the particles were prepared at a total flow rate (TFR) of 2 mL/min and a flow rate ratio (FRR) of 1:5 organic to water phase. To investigate the effect on drug loading of the different microfluidic parameters, the TFR (2–12 mL/min) and the FRR (1:1–1:10) were varied.

For both preparation techniques, immediately after preparation, particles were diluted to 20 mL with Millipore water and purified and concentrated through ultracentrifugation with a 30-kDa molecular weight cutoff Microsep advance centrifugal device (Pall corporation, NY, USA) at 959 x *g* for 20 min. Purification from free or adsorbed drug was performed through thoroughly washing the NPs with Millipore water and ultracentrifugation, as described above (2×).

### Particle Characterization

The particle size was determined through dynamic light scattering (DLS) using a ZetaSizer Nano ZS. The device was equipped with a 633 nm He-Ne laser at a backscatter angle of 173° (Malvern Instruments GmbG, Lappersdorf, Germany). Measurements were performed at 25°C using Millipore water as dispersing medium and a NP concentration of 1 mg/mL. The measurement position was set at 4.65 mm and the data was recorded with Malvern Zetasizer software 7.11 (Malvern Instruments, Worcestershire, United Kingdom).

The particle PEG content was determined using a colorimetric iodine complexing assay (30) as previously described by our group (29).

The NP mass concentration was determined gravimetrically after lyophilization and the PFD content was measured photometrically after particle disruption in ACN at 300 nm using a FLUOStar Omega microplate reader (BMG Labtech, Ortenberg, Germany). The NP molar concentration (PNC) was calculated using Eq. 8 where *m* is the NP mass determined gravimetrically after lyophilization, *ρNP* is the density of the particles (1.25 g/cm^3^) (31), *dh* is the hydrodynamic diameter of the NPs determined through DLS, *N*_*A*_ is the Avogadro number, and *V* the volume of the samples.

8 $$ PNC=\frac{m}{\rho NP\ \frac{4}{3}\ \pi {\left(\frac{dh}{2}\right)}^3}\ \mathrm{x}\frac{1}{N_A\ V} $$

The encapsulation efficiency (EE) was determined using Eq. 9*,* where *m*_*E*_ is the quantified mass of entrapped drug, and *m*_*T*_ is the total mass of drug added initially to the formulation.

9 $$ EE\ \left(\%\right)=\frac{m_E}{m_T}\ x\ 100 $$

The loading capacity (LC) was determined through Eq. 10*,* where M_T_ is the total mass of the particle formulation.

10 $$ LC\ \left(\%\right)=\frac{m_E}{M_T}\ x\ 100 $$

The number of drug molecules per NP was calculated by dividing the molar concentration of the drug by the molar concentration of the NPs determined as described above.

### Determination of the Spatial Restriction of PFD Loading

To determine the number of molecules that fit inside a NP, the volume of the particle core was estimated. To that end, the mean NP size was used as a starting point. For the calculation it was considered that the PLA is located at the core-shell interface and that the PLGA and PEG form the core and shell, respectively. To estimate the core size, first the conformation of the PEG on the particle surface was investigated using the Flory radius (R_F_) (32). When the distance between polymer chains on a particle surface is smaller than the R_F_, they assume an extended brush conformation. Otherwise, they take a folded mushroom configuration. The R_F_ can be calculated using Eq. 11, where *α* is the length of a PEG monomer (0.278–0.358 nm) (33) and *N* is the number of monomers in one molecule (each monomer has the molecular weight of 44 g/mol).

11 $$ {R}_F=\alpha {N}^{\frac{3}{5}}\kern0.5em $$

The surface (S) that is taken by the polymers on the particle surface can be calculated after Gref *et al.* (34) using Eq. 12. *M*_*PEG*_ represents the molecular weight of the PEG, and *f* is the mass fraction of PEG in the PEG-PLA blends. The *S* can be used to determine the distance between polymers (*D*) on the particle surface using Eq. 13 (34).

12 $$ S=\frac{6\ {M}_{PEG}}{dh\ {N}_A\ f\ \rho NP} $$

13 $$ D=2\ {\left(\frac{S}{\uppi}\right)}^{\frac{1}{2}}\kern0.5em $$

The length in nm of a PEG brush can be calculated by multiplying the monomer length *α* = 0.35 nm by *N*. This value was subtracted twice from the *dh* of the NP to obtain the diameter of the particle core (*d*_*c*_). The volume of the core (*V*_*core*_) was calculated assuming a spherical shape after Eq. 14, where *r*_*c*_ is the calculated radius of the particle core (*d*_*c*_/2).

14 $$ Vcore=\frac{4}{3}\pi {r}_c^3 $$

The estimation of the molecular volume of PFD (*V*_*drug*_ *= 0.2 nm*^*3*^) was conducted using the group contribution methods after Fedors (25). The number of drug molecules that are able to fit inside a single particle core were calculated through Eq. 15*,* assuming the maximal packing efficiency of poly-sized spheres (90%) (35), a spherical molecule shape and an even drug distribution among all the particles.

15 $$ Drug\ molecules\  per\  NP=\frac{V_{core}}{V_{drug}}\ x\ 0.9 $$

In order to prepare particles with increasing PLGA core sizes, the PLGA content of the formulations was increased. Particles with a PEG-PLA to PLGA mass ratio of 70:30, 60:40, 50:50 and 40:60 were prepared to a total final polymer concentration of 10 mg/mL, as described above. NPs were prepared through bulk nanoprecipitation and an initial PFD mass of 25 μg, as described above.

### Co-Encapsulation of LA

For the co-encapsulation of LA and PFD, the NPs were prepared as described above. LA and PFD were simultaneously added to the organic polymer mixture prior to particle preparation. The PFD to LA molar ratio was varied to determine the ideal particle composition. Additionally, the initial PFD amount was set to 2 mg and LA in a molar excess (ranging from 0.1 to 3.5) was added to the different formulations. As a control, NPs only encapsulating LA at the same concentrations added to the PFD-containing formulations were prepared. As an additional control the molar excess of LA added was replaced by the same molar excess of PFD. The PFD or LA content in the formulations was determined photometrically after particle lyophilization and disruption in ACN at 300 or 334 nm, respectively, using a FLUOStar Omega microplate reader (BMG Labtech, Ortenberg, Germany). The EE and number of drug molecules per particle was determined as described above.

### ***In Vitro*** Release Studies

The *in vitro* release of PFD was assessed through the dialysis bag method. To that end, NPs containing PFD (NP-PFD), or PFD and LA (NP-PFD/LA) were prepared at a 70:30 PEG-PLA to PLGA mass ratio and purified as described above. An initial PFD and LA mass of 2 mg and 4.5 mg, respectively, were used. The samples were adjusted to a final volume of 2 mL (60 mg/mL NPs) and placed in a 3.5-5 kDa molecular weight cut off Spectrum™ Spectra/Por™ 3 RC Dialysis Membrane (Spectrum Laboratories, Inc., Rancho Dominguez, CA, USA). The dialysis membrane was introduced in a 35 mL glass vial containing 27 mL of DPBS (pH 7.3) under sink conditions. Vials were kept in a 37°C shaking water bath and 0.5 mL samples were taken at different time points and replaced with fresh pre-warmed buffer. As a control, particles with no encapsulated drug (NPMeO), and free drug (PFD, 2.2 mg) were used. The PFD concentration was quantified at 300 nm using a FLUOStar Omega microplate reader (BMG Labtech, Ortenberg, Germany).

### Statistical Analysis

Statistical analysis was performed using GraphPad Prism Software 6.0. Student’s t test (Fig. 3 and Fig. 5a) was employed to evaluate statistical significance. Levels of statistical significance and “n” numbers for each experiment are indicated in the text and figure legends.

## Results and Discussion

### Thermodynamic Considerations for PFD Encapsulation

A frequent approach to estimate the compatibility of a drug in a polymer is the calculation of their thermodynamic interactions. This approach has been used by several authors to select the most suitable polymers for encapsulating a certain drug or retroactively explain experimental outcomes (26,27,36–38). Here, we assessed the thermodynamic miscibility of PFD and LA with either PEG, PLA or PLGA to determine the suitability of our particle system for the selected drug(s). As depicted in Eq. 16, the thermodynamic miscibility of two substances is given by the free energy of mixing (ΔG_M_), where ΔH_MT_ and ΔS_M_ are the enthalpy and entropy of mixing, respectively. If the ΔG_M_ is negative, the two substances are considered mutually soluble.

16 $$ \Delta  {G}_M=\Delta  {H}_{MT}-T\Delta  {S}_M $$

The mixing enthalpy (ΔH_M_) per volume unit for each polymer-drug can be used as an indicator for the miscibility of two substances. It is given by Eq. 5 after Hildebrand (26). For its determination the volume fractions and the solubility parameters of the drug and polymer, Φ_1_ and Φ_2_ and δ_1_ and δ_2_ respectively, are considered.

The solubility parameters for some common drugs and polymers are reported in the literature (22,23). Otherwise they can be calculated after van Krevelen and Nijenhuis (24) using Eq. 4 by combining the contribution of the partial solubility parameters associated with the dispersion forces (δ_d_), the polar interactions (δ_p_) and the hydrogen bonding components (δ_h_). The total- and partial solubility parameters of PFD, LA and the particle-forming polymers are depicted in Table I.

**Table I:** Partial and Total Solubility Parameters for the Drugs (PFD and LA) and the Particle-Assembling Polymers

Component	δ_d_	δ_p_	δ_h_	δ
PFD^*^	20.5	9.4	7.7	23.8
LA^*^	22.9	2.9	10.2	25.2
PEG^#,^ (22)	20.3	9.6	6.0	24.0
PLA^#,^ (22)	19.8	4.0	6.7	22.0
PLGA^#,^ (23)	17.4	9.1	10.5	22.3

Obtained from literature#, calculated*

The miscibility of two components is promoted when their solubility parameters have similar values and thus, ΔH_MT_ tends to 0. Therefore, the differences among solubility parameters of drug and polymer (Δδ_,_ Δδ_d,_ Δδ_p_, and Δδ_h_) can also be used to predict miscibility (37) (Table II).

**Table II:** Calculated Differences Between the Solubility Parameters or Polarity of PFD or LA and the Particle-Forming Polymers

Particle component	PFD				LA			
	Δδ_d_	Δδ_p_	Δδ_h_	Δδ_t_	Δδ_d_	Δδ_p_	Δδ_h_	Δδ_t_
PEG	0.2	- 0.2	1.7	- 0.2	2.6	- 6.7	4,2	1.2
PLA	0.7	5.4	1.0	1.8	3.1	- 1.1	3.5	3.2
PLGA	3.1	0.3	- 2.8	1.5	5.5	- 6.2	- 0.3	2.9

However, as Liu *et al.* pointed out, Eq. 5 does only take into consideration dispersion forces (27). As an alternative, they proposed the determination of ΔH_M_ taking into account all forces of the interaction by using the partial solubility parameters, as depicted in Eq. 6.

The most frequently used indicator to predict the miscibility of drug molecules with different polymers is the Flory-Huggins interaction parameter (χ_sp_) (8). It is given by the Hildebrand-Scatchard equation (Eq. 7), which takes into account the total solubility parameters (δ) of the drug and polymer in combination with the molar volume of the drug (V_drug_). A complete miscibility is achieved when χ_sp_ is <0.5 (24) which requires a very near match of both solubility parameters of drug and polymer. The closer the χ_sp_ is to zero, the more favorable interactions between the two components, and thus the better compatibility of drug and polymer.

**Fig. 2:**
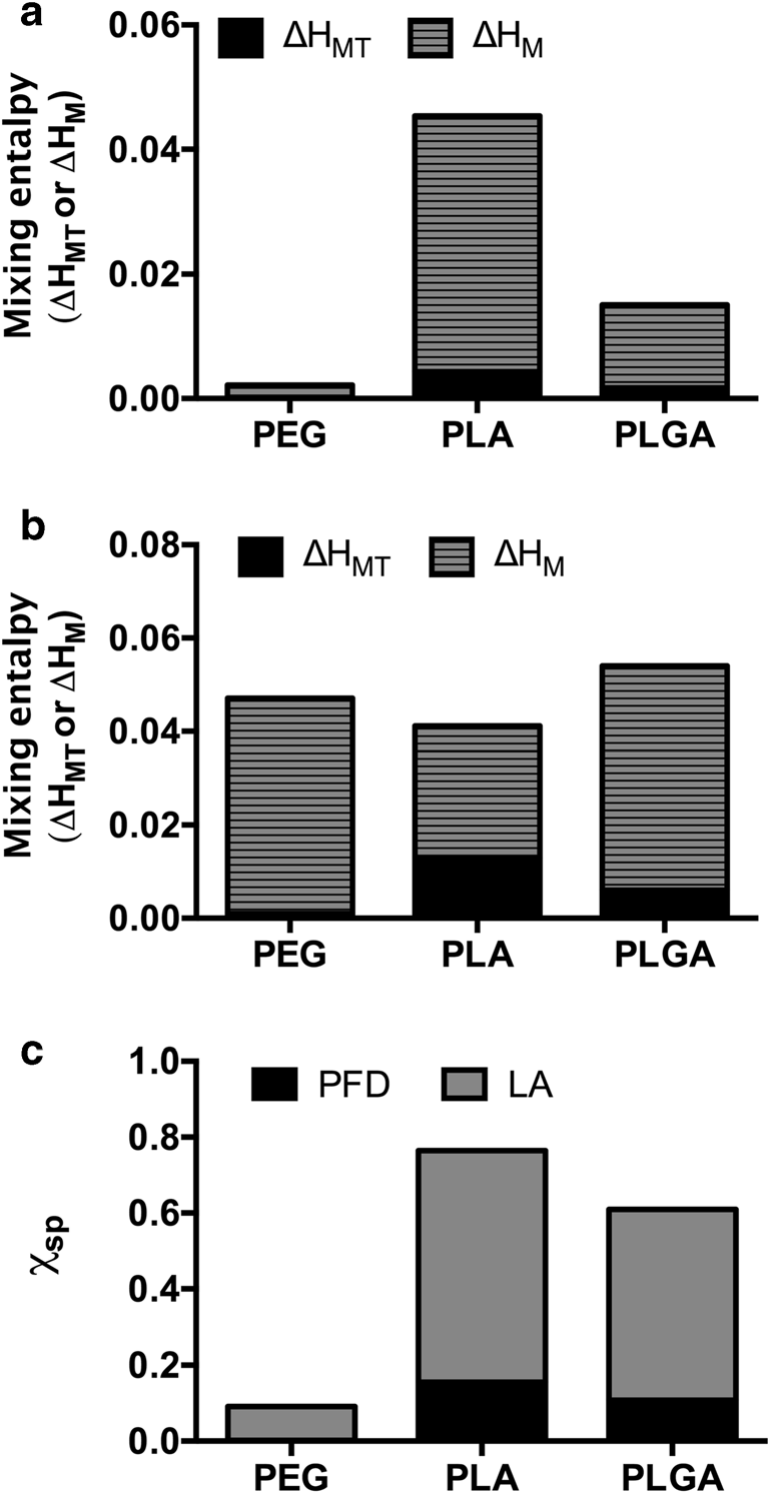
Prediction of the drug-polymer miscibility. Mixing enthalpy of the particle-assembling polymers and (**a**) PFD or (**b**) LA. (**c**) Flory-Huggins interaction parameter.

With the determined solubility parameters (δ_,_ δ_d,_ δ_p_, and δ_h_) (Table I) and their differences (Δδ_,_ Δδ_d,_ Δδ_p_, and Δδ_h_) (Table II), we calculated the mixing enthalpy (ΔH_MT_ or ΔH_M_), and χ_sp_ in order to predict the miscibility of our drugs with the particle system (Fig. 2).

Comparing the total solubility parameters (δ) (Table I), it is evident that all substances (drugs and polymers alike) have very similar values ranging between 22 and 25 MPa^1/2^. This indicates a very good match for the selected drug and polymers, which is confirmed by the values of ΔH_MT_ and ΔH_M_ of the PFD- or LA-polymer mixtures (Fig. 2). As pointed out by Liu *et al.* (27), the absolute values for ΔH_MT_ and ΔH_M_ differed. Furthermore, for the case of LA, a complete opposite tendency was seen (Fig. 2b). The ΔH_MT_ predicted a higher miscibility for PEG > PLGA > PLA, whereas the ΔH_M_ anticipated a better miscibility with PLA > PEG > PLGA. Nevertheless, the enthalpy values for all mixtures were very close to zero predicting good miscibility from a thermodynamic point of view.

As the most common used parameter to predict drug-polymer compatibility is the χ_sp_, we also compared these values for the polymers composing our NPs. As depicted in Fig. 2c, the χ_sp_ values predict total miscibility of PFD with all the polymers forming the particle with a predilection to PEG > PLGA > PLA. However, for LA it indicates low miscibility with PLGA and PLA, as χ_sp_ < 0.5 only for PEG, disagreeing with the other estimated parameters. Overall, as suggested previously by other authors (27), the different parameters predicting drug-polymer miscibility varied in terms of the polymer which showed the highest miscibility. Nevertheless, except the χ_sp_ for the LA-PLA or LA-PLGA mixture, all the obtained values indicated thermodynamically favorable miscibility. In general, the highest miscibility for PFD is predicted to be with PEG, followed by PLGA and PLA (Fig. S1A and Table SI). For LA the same trend in miscibility is to be expected (Fig. S1B and Table SI).

As all determined parameters unanimously predict that PFD is very compatible with all the particle-forming polymers, we determined that the block copolymer PEG-PLA/PLGA particle system is an appropriate candidate for its encapsulation.

### Influence of the Nanoprecipitation Technique on PFD Loading

**Fig. 3:**
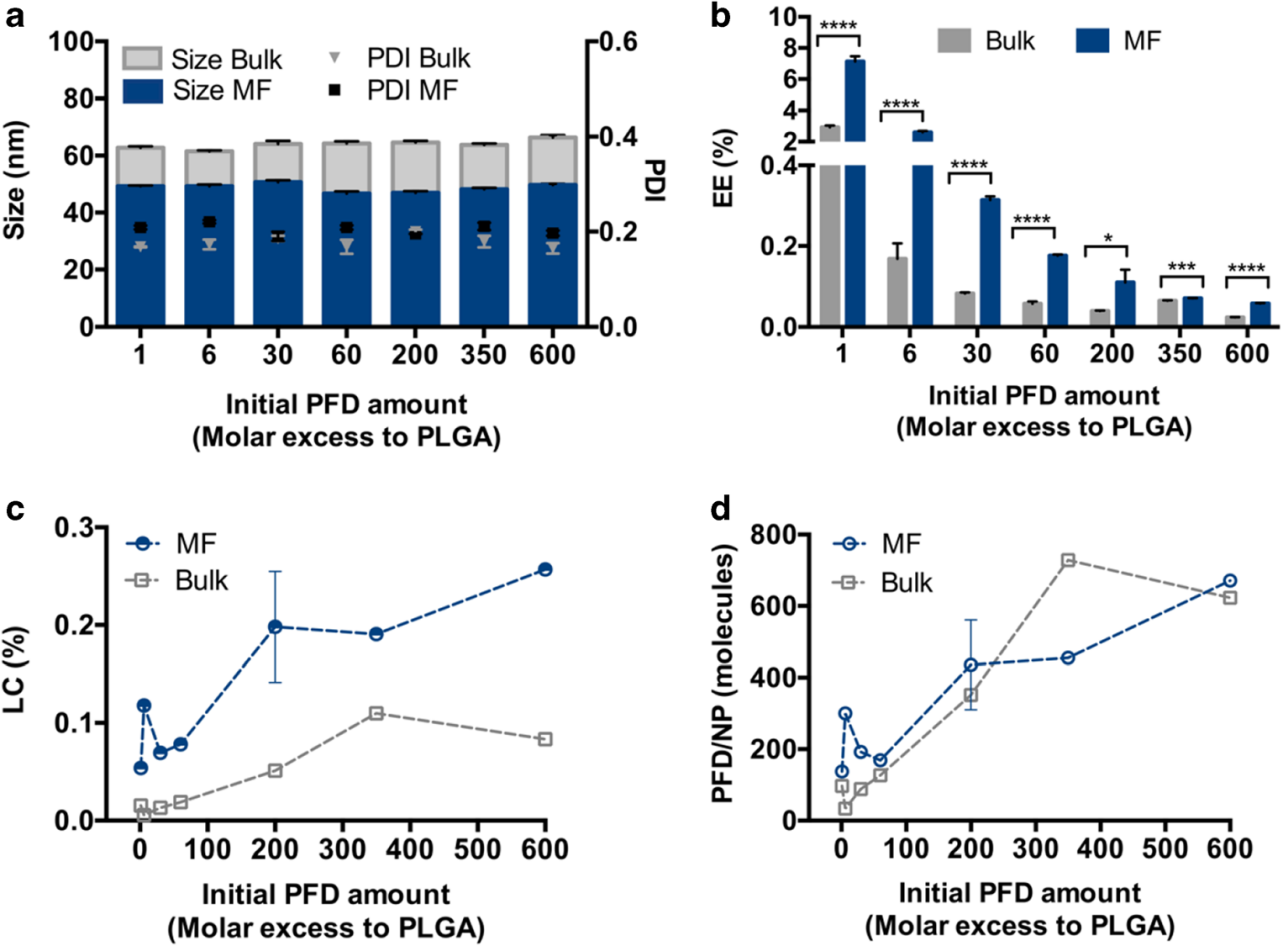
PFD encapsulation in block copolymer NPs through bulk and MF nanoprecipitation. Particle characterization in terms of (**a**) size and PDI, (**b**) Encapsulation Efficiency (EE) (**c**) Loading Capacity (LC) and (**d**) number of PFD molecules per particle. Results are shown as mean ± SD of at least *n* = 3 measurements. Levels of statistical significance are indicated as *p ≤ 0.005, ***p ≤ 0.001, and ****p ≤ 0.0001.

In order to experimentally investigate the encapsulation of PFD in PEG-PLA/PLGA block copolymer NPs, we evaluated two different manufacturing techniques, bulk or MF nanoprecipitation (Fig. 3). To that end, PFD-polymer mixtures in ACN were precipitated into a water phase, either manually or with the assistance of a microfluidic device. The initial PFD addition was varied to determine its influence on the drug loading. The resulting particles were homogenous in terms of size with narrow polydispersity indexes (PDI) (Fig. 3a) independent of the amount of drug added to the formulation. As frequently observed, MF manufacturing rendered particles about 15 nm smaller (~ 50 nm) compared to bulk nanoprecipitated particles (~ 65 nm). This is due to the fact that the MF mixing is faster than the time the particles need to nucleate, which does not occur during bulk nanoprecipitation (39).

For both methods an increase in initial drug concentrations reduced the EE (Fig. 3b), which can be partially explained by an increase in mixing enthalpy with increasing volume fractions of drug (Fig. S2). Interestingly, MF manufacturing achieved significantly higher EE, a phenomenon also described by other authors (40,41). As an additional advantage, MF preparation allows for a precise control over the manufacturing parameters, such as the TFR (Fig. S3) and the FRR (Fig. S4) which, in turn, permit drug loading optimization and a decrease in batch to batch variations. Furthermore, the resulting particles have the ideal size (~ 50 nm) for optimal NP-cell interaction (42) (Fig. S5).

Despite the decrease in efficiency of the process with higher PFD amounts, the LC (Fig. 3c) and number of drug molecules per particle (Fig. 3d) increased with larger initial PFD additions. This is frequent for particles entrapping the drug during NP preparation (39), and is probably promoted in this particular case by the water solubility of PFD (~ 2 mg/mL). Interestingly, both methods achieved a comparable number of PFD molecules per particle, which is probably due to a larger number of smaller particles being produced by MF compared to bulk nanoprecipitation.

However, the low number of drug molecules that were being incorporated into the particle system (under 1000 molecules per NP) was quite surprising, considering the high predicted miscibility of PFD with the particle-forming polymers (Fig. 2). By those calculations, PFD displayed a very high compatibility with PEG. Therefore, part of the drug may end up in the particle’s shell. However, during NP purification, this non-core-bound drug is washed out through the several purification steps, promoted by the water solubility of PFD, causing a lower than expected EE.

Overall, we could demonstrate that PFD encapsulation in block copolymer NPs is feasible by nanoprecipitation. Comparing both manufacturing techniques, it is apparent that MF is superior. Not only did it lead to a significantly higher EE than bulk nanoprecipitation, but this could be further enhanced up to 40% by adjusting the manufacturing parameters (Fig. S3 and S4). More so, a higher cellular uptake was achieved by MF-nanoprecipitated particles (Fig. S5) which is highly relevant for *in vitro* and *in vivo* applications.

### Spatial Limits on the Drug Encapsulation

**Fig. 4:**
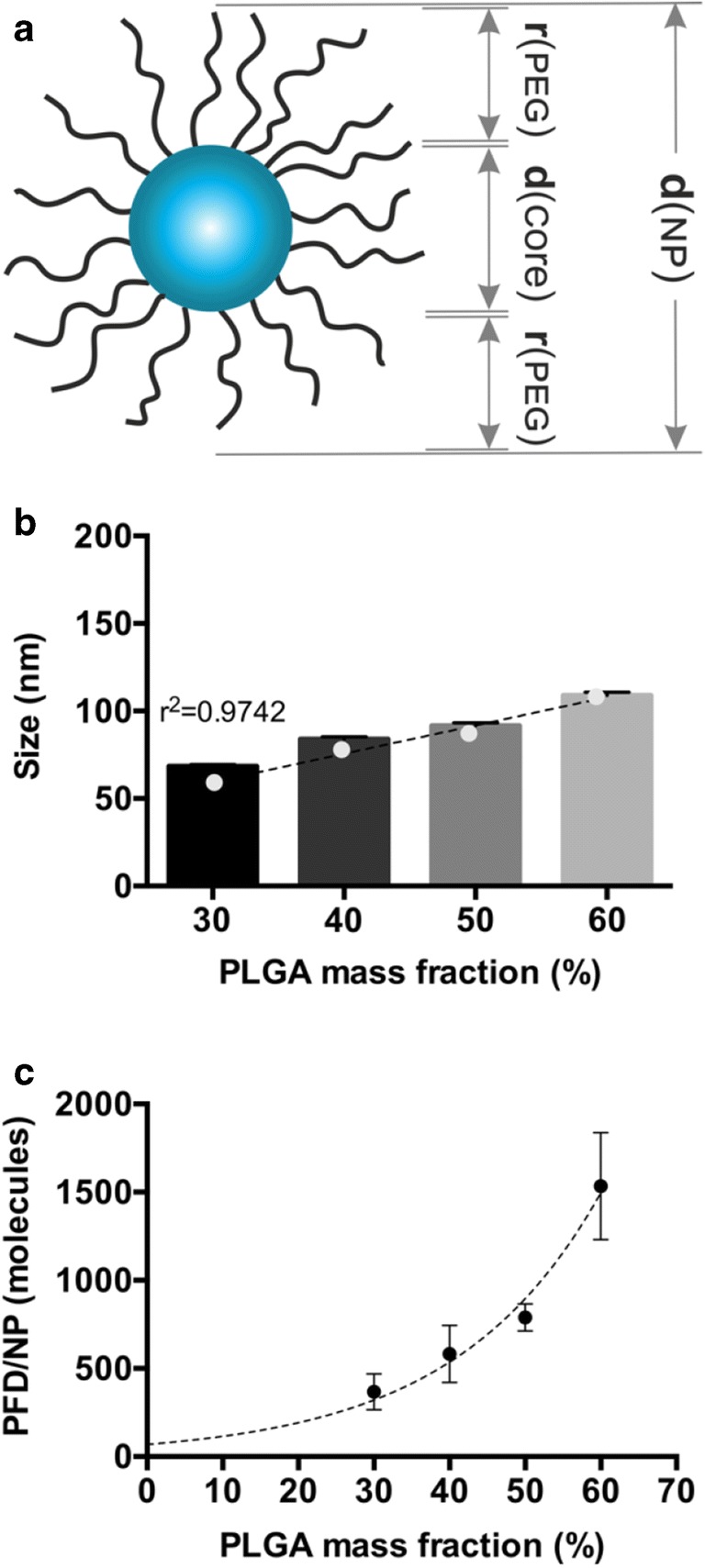
Core volume-dependent PFD encapsulation. (**a**) structure of a block copolymer NP. d(NP): particle diameter; r(PEG): PEG-brush radius; d(core): particle core diameter. (**b**) NP size and (**c**) PFD molecules per NP with increasing PLGA mass fraction. Results are shown as mean ± SD of at least n = 3 measurements. Data in (**b**) and (**c**) are fitted with a linear and an exponential growth equation, respectively.

Due to the low number of PFD molecules being incorporated in our particles (Fig. 3), despite the high predicted miscibility with the selected polymers, we decided to estimate the number of drug molecules that would actually fit inside a single particle’s core (Fig. 4). For our determination we considered a particle structure as depicted in Fig. 4a, where the PEG composes the shell, the PLGA forms the particle core, and the PLA is located at the core-shell interface. Given that after NP manufacturing, the particles are purified from drug that is free or entangled in the PEG shell through various washing and centrifugation steps, we considered that the particle-bound drug would mostly be located in the PLGA core. We speculated that the spatial constriction given by the core size would determine the number of PFD molecules that can be fitted inside the particle.

To determine the size of the core, the length of the PEG brush was calculated and subtracted from the overall particle diameter (~ 65 nm). To that end, we firstly determined the conformation of the PEG shell on the particle corona. For our NPs prepared at a 70:30 PEG-PLA to PLGA mass ratio, the PEG assumes an extended brush conformation. This corresponds to a length of approximatively 30 nm (for a PEG with a molecular weight of ~ 4800 Da). This would leave a particle core of about 5 nm in diameter, which would allow for the encapsulation of about 200 PFD molecules per NP, assuming spherical shapes and 90% packing efficiency. It needs to be noted that this calculation does not take into consideration the space occupied by the core-forming PLGA itself. However, the value is in the same size-range of that was experimentally determined (Fig. 3).

Therefore, we hypothesized that the number of encapsulated molecules per particle could be improved by increasing the size of the PLGA core. To confirm these assumptions, particles with an increase in PLGA mass ratio were prepared. From the initial PEG-PLA to PLGA 70:30 mass ratio, we raised the PLGA fraction to 60:40, 50:50 and 60:60. As expected, the NPs increase in size in a linear manner from 60 to 120 nm (Fig. 4b). However, for all the formulations the PEG assumed a brush conformation (Table SII), indicating that the size increase was core-derived (Fig. S6). More importantly, the number of PFD molecules per particle increased exponentially (Fig. 4c), therefore confirming our theory that there is a spatial restriction for drug loading in a particle.

### Co-Encapsulation of LA and PFD in Block Copolymer NPs

Even though increasing the size of the PLGA core dramatically enhanced the number of PFD molecules per NP, for certain applications this may not be a feasible option. More so, the number of PFD molecules per NP did not reach the extent that would be expected from the linear size increase (Fig. S7). This can be explained, on the one hand, by the volume that the PLGA takes up in the core by itself and which was not considered for the calculations. On the other hand, the high affinity of PFD to PEG may limit its core localization and promote a partial distribution among the NP shell. This PEG-associated PFD is removed during particle purification due to water solubility of the drug (2 mg/mL). However, a PEG shell is highly necessary for the *in vivo* applications of NPs, as it reduces protein adsorption (29,34) and increases their blood residence (43).

As an alternative approach, we hypothesized that inclusion of an additional drug molecule, also compatible with PEG but highly water insoluble, would increase the number of PFD molecules per NP by limiting the PFD diffusion to the aqueous phase.

As a candidate we selected LA. It has a very poor water solubility (0.2 mg/mL), which is 10-times lower than for PFD (2 mg/mL). Additionally, as determined by the miscibility prediction (Fig. 2), it is compatible with the particle forming polymers, especially with PEG. More so, at a therapeutic level it may show complementary effects to PFD as they both demonstrate positive antioxidative effects in doxorubicin-induced cardiac and renal toxicity (44) and oxidative liver damage (45).

**Fig. 5:**
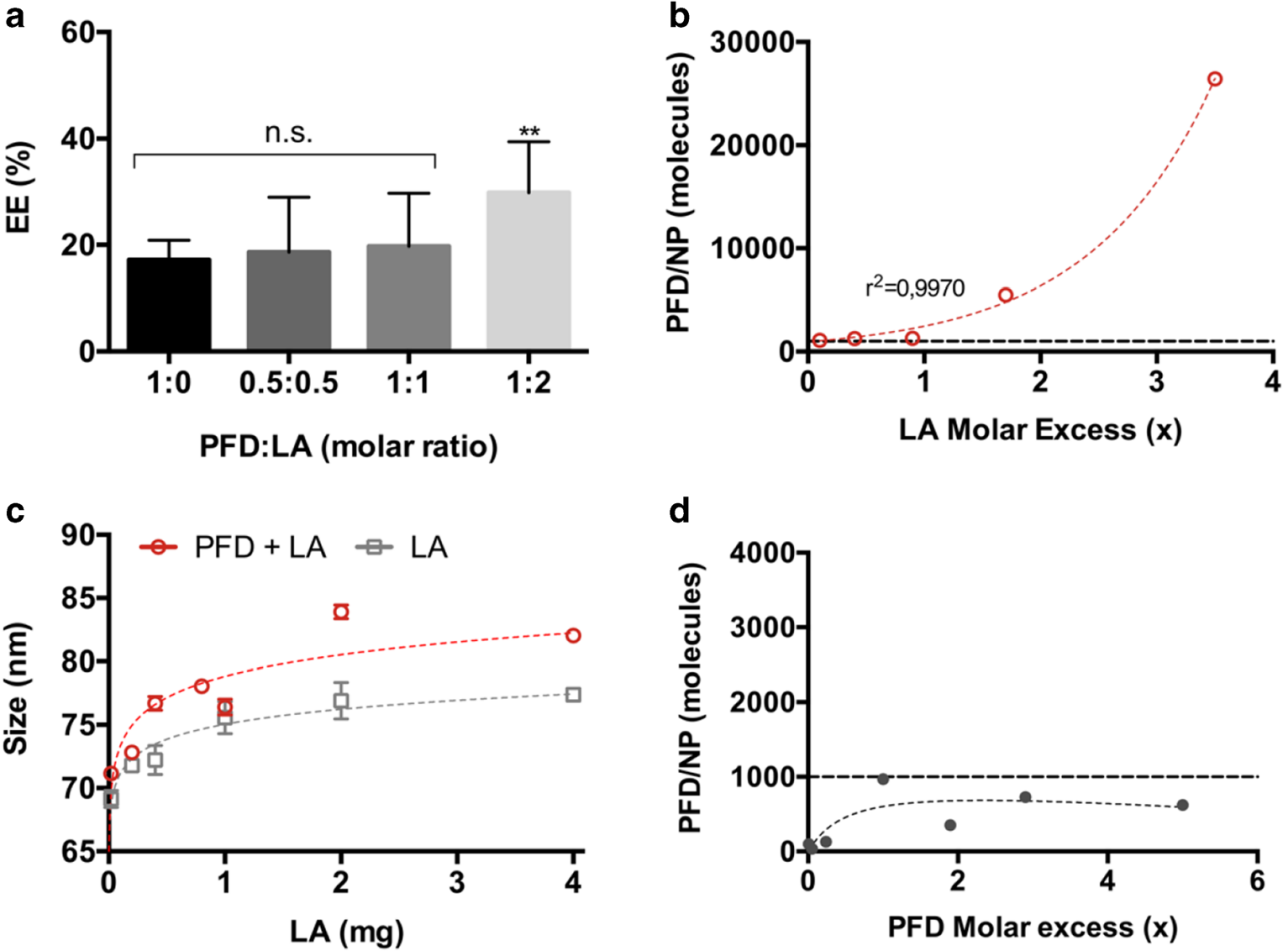
Co-encapsulation of LA and PFD in block copolymer NPs. (**a**) NPs prepared with varying PFD to LA molar ratios. (**b**) PFD molecules per particle and (**c**) size of NPs prepared with increasing LA amounts. (**d**) Maximum number of PFD molecules per particle at increasing initial PFD amounts. Results are shown as mean ± SD of at least n = 3 measurements. Levels of statistical significance are indicated as **p ≤ 0.01 and *p ≤ 0.05 compared to NPs without LA, n.s.: non-significant. Data in (**b**) are fitted with an exponential growth equation. Data in (**c**) and (**d**) are fitted with a saturation curve.

To test our hypothesis, we prepared NPs encapsulating both PFD and LA (Fig. 5). Initially we combined different PFD and LA ratios and examined the EE. As depicted in Fig. 5a, a LA molar excess significantly increased the EE from 20% to 40%. We then maintained a constant PFD concentration and varied the formulation’s LA content. Interestingly, an exponential increase/decrease in PFD molecules per NP was observed as LA content was increased/lowered (up to 25,000 PFD molecules/NP) (Fig. 5b). When comparing the size of particles only incorporating LA with the ones encapsulating LA and PFD, there was only a 10 nm size difference (Fig. 5c). Furthermore, the number of PFD molecules per NP reached with increasing LA addition could not be achieved by just increasing the initial PFD added to the formulations (Fig. 5d), which reached a maximum of about 1000 molecules per particle (dashed line in Fig. 5b and d). This rising number of PFD molecules per NP can be explained by a decrease in mixing enthalpy with increasing amounts of LA, which may promote the miscibility of PFD with the particle-forming polymers (Fig. S8). Additionally, the lower water solubility of LA and its interaction with PFD may promote a stronger particle binding of the latter. This is reinforced by the fact that the single encapsulation of LA, reaches a higher number of molecules per NP (Fig. S9) compared to the individual PFD encapsulation.

**Fig. 6:**
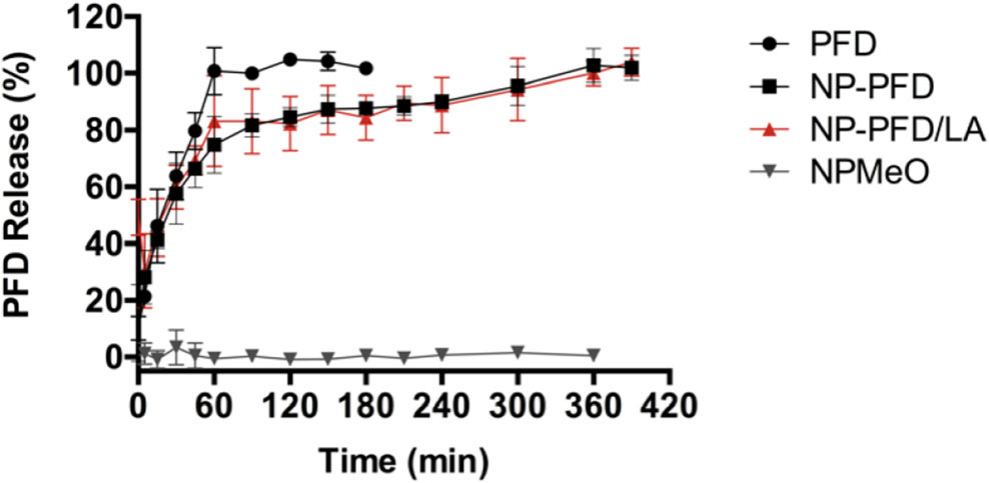
Release profile of PFD from block copolymer NPs. PFD: Free drug; NP-PFD: PFD-loaded NPs; NP-PFD/LA: PFD- and LA-loaded NPs; NPMeO: control, drug-free NPs. Results are shown as mean ± SD of at least *n* = 3 measurements.

However, the co-encapsulation of an additional drug molecule can raise concerns about its interference with the release profile of the original drug. Therefore, we investigated the PFD *in vitro* release from the different NP formulations using the dialysis bag method. To that end, NPs containing PFD (NP-PFD) or PFD and LA (NP-PFD/LA) were compared. As controls, the release profiles under the same conditions of drug-free particles (NPMeO) and free drug (PFD) were evaluated. As depicted in Fig. 6, after 1 h all of the free-drug was released from the dialysis membrane. At that time point, about 70% of the PFD was released from the NPs. The remaining 30% of the particle-bound drug was released very slowly over the course of the next 5 h. This fast release is probably due to the large surface area to volume ratio resulting from the small particle size. Additionally, the water-solubility of PFD may boost this effect. However, these results are in line with the release of other hydrophilic drugs prepared through nanoprecipitation, such as procaine hydrochloride from PLGA NPs (4). Comparing the release profiles of NP-PFD and NP-PFD/LA it becomes apparent that both particle formulations show the same release curve. Therefore, we can conclude that the LA incorporation has no detrimental effects on the PFD release (Fig. 6).

## Conclusion

In this study we systematically examined the encapsulation of PFD, a water-soluble drug lacking ionizable groups, in block copolymer NPs through nanoprecipitation. We determined different thermodynamic parameters which predicted a good miscibility of the drug with the particle-forming polymers (PEG, PLA and PLGA) (Fig. 2). We showed that the calculation of different parameters yielded slight differences among their predictions (Fig. S1), which is in agreement with previous studies. More so, even though useful for selecting appropriate polymers for the drug encapsulation, they cannot be exclusively considered. We demonstrate that spatial constrictions regarding the particle’s core size can also limit the number of molecules that can be encapsulated in polymer NPs (Fig. 4) and by modulating them the number of encapsulated molecules can be increased. We experimentally assessed the EE, LC and number of encapsulated drug molecules per particle using two common nanoprecipitation techniques (Fig. 3). Among them, we were able to demonstrate that MF manufacturing achieves significantly higher drug loading with the additional benefit of precise adjustment of the process parameters (Fig. S3 and S4) and enhanced NP-cell interactions (Fig. S5).

Lastly, we demonstrated how the encapsulation of an additional drug, LA, with similar polymer miscibility but lower water solubility is able to exponentially increase the number of PFD molecules per NP (Fig. 5) without affecting the drug’s release profile (Fig. 6).

Overall our study demonstrates that the encapsulation of a water-soluble drug without ionizable groups is feasible when taking into consideration and adjusting the limits due to size constrictions and appropriately selecting the manufacturing method (MF vs bulk nanoprecipitation). More so, by considering the co-encapsulation of an additional appropriate molecule an additional increase in drug loading can be achieved.

## Electronic supplementary material

ESM 1 (DOCX 14121 kb)

## Abbreviations

χ_sp_: Flory-Huggins interaction parameter
FRR: Flow rate ratio
LA: α-lipoic acid
PEG: Poly(ethylene glycol)
PFD: Pirfenidone
PLA: Poly(lactic acid)
PLGA: Poly(lactic-co-glycolic acid)
MF: Microfluidic
ΔH_MT_: Mixing enthalpy obtained from the total solubility parameters
ΔH_M_: Mixing enthalpy obtained from the partial solubility parameters
NPs: Nanoparticles
δ: Solubility parameter
TFR: Total flow rate
