# Supplementary material for: Thermodynamic, Spatial and Methodological Considerations for the Manufacturing of Therapeutic Polymer Nanoparticles
Source: Pharm Res. 2020 Feb 24;37(3):59. doi: 10.1007/s11095-020-2783-4 (PMC7040083; doi:10.1007/s11095-020-2783-4)
Supplement: Supplementary file 1 — (DOCX 14121 kb) [file 11095_2020_2783_MOESM1_ESM.docx]

**Supplementary Information**


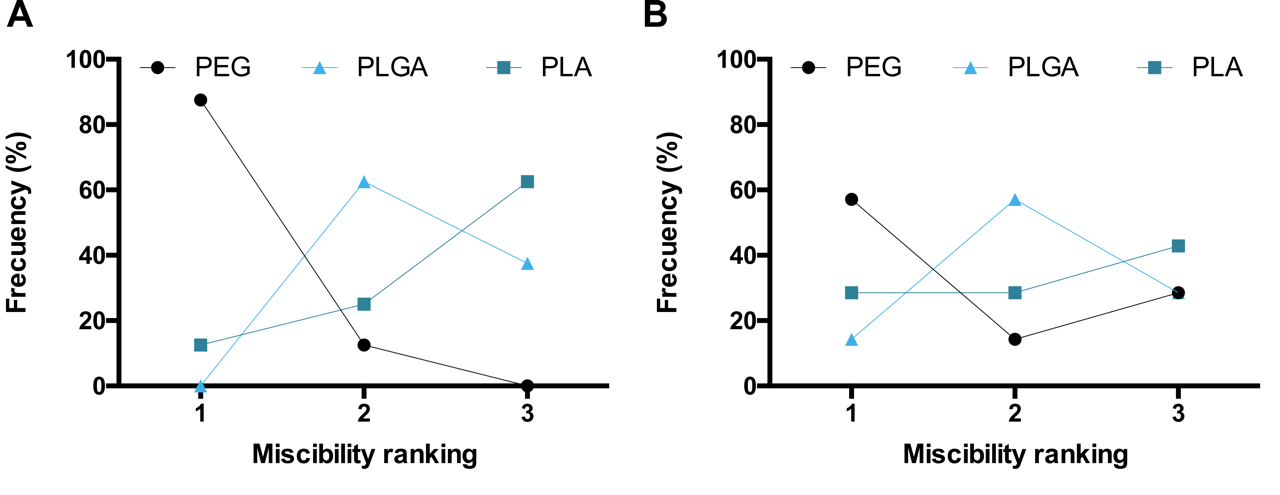


**Figure S1.** Ranking of the (A) PFD- and (B) LA polymer miscibility determined by the calculation of 7 predicting parameters (Δδ_,_ Δδ_d,_ Δδ_p_, Δδ_h_, ΔH_MT_, ΔH_M_, and χ_sp_). The majority of the calculated parameters indicate the highest miscibility of PFD and LA with PEG (80% or 50% of the considered parameters, respectively), followed by PLGA (65% or 60%) and PLA (65% or 45%). For each parameter´s individual miscibility prediction please see Table SI.

**
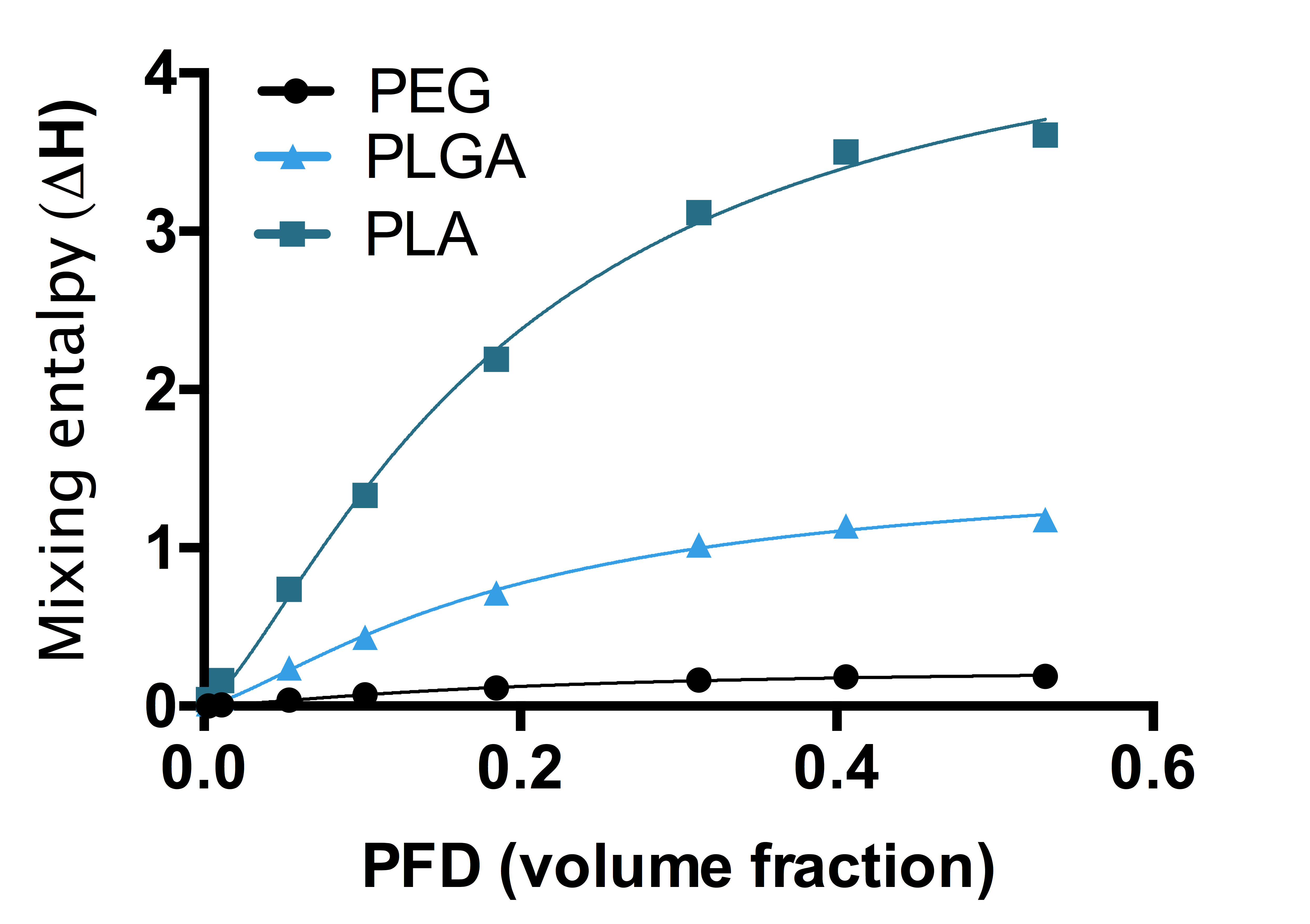
**

**Figure S2**. Mixing enthalpy of PFD with PEG, PLA or PLGA at increasing drug volume fractions. At increasing PFD volume fractions, the miscibility with the different particle-assembling polymers decreases.

**
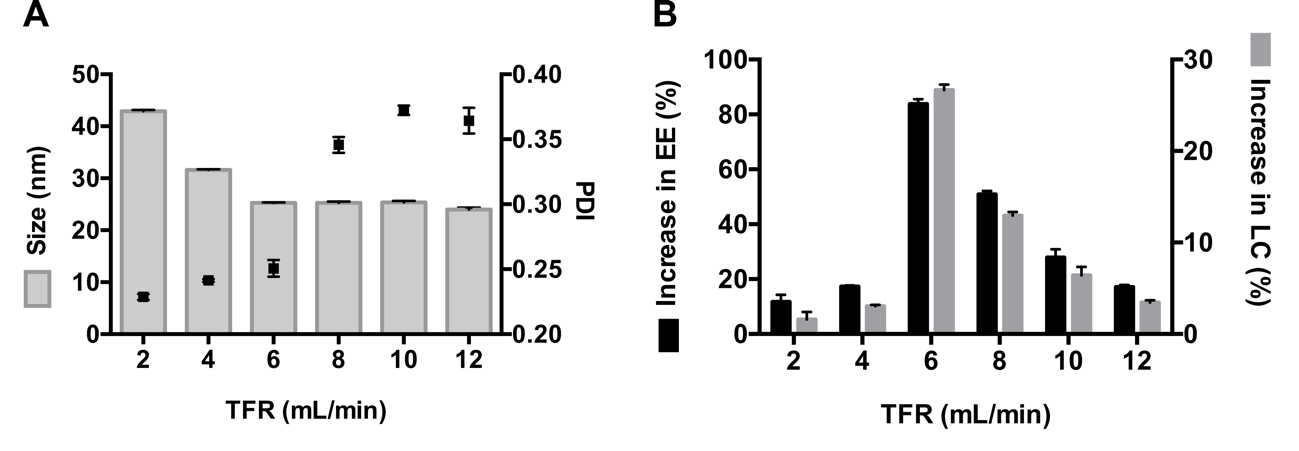
**

**Figure S3.** Effect of the total flow rate (TFR) during MF manufacturing on (A) particle characteristics (size and PDI) and (B) increase in EE and LC in block copolymer NPs. Increasing the TFR decreases the overall particle size. However, at TFR > 8 mL/min, the PDI of the resulting NPs drastically increases, due to particle instability and aggregation. A TFR of 6 mL/min achieves the highest enhancement of the EE compared to NPs prepared via bulk nanoprecipitation. A deviation from this TFR results in a lower enhancement of the EE.

**
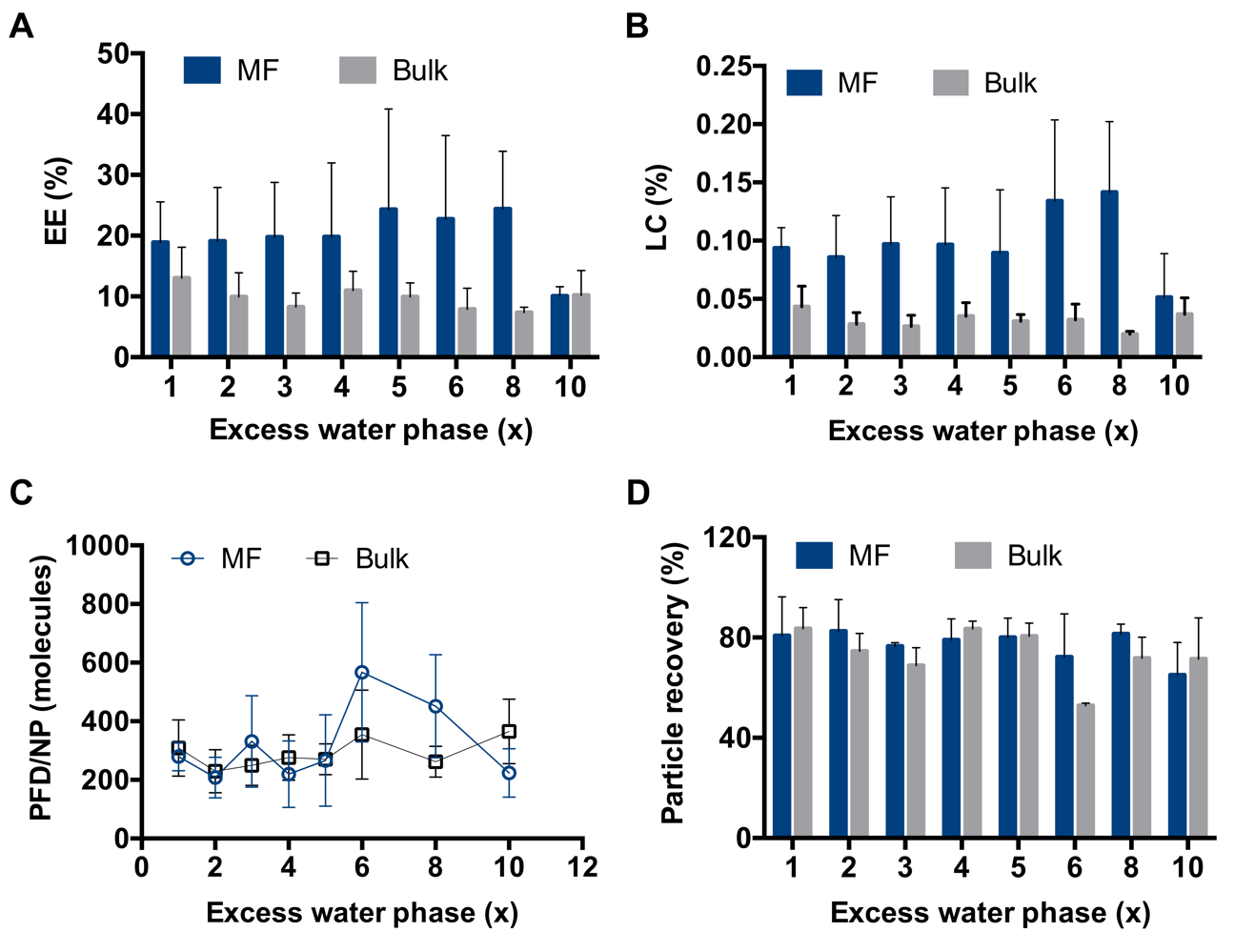
**

**Figure S4**. Influence of the flow rate ratio (FRR) during nanoprecipitation on PFD loading in block copolymer NPs. (A) EE, (B) LC, (C) number of molecules per particle, and (D) particle recovery after preparation and purification. Particles were prepared *via* bulk nanoprecipitation or MF manufacturing (TFR of 2 mL/min). Independent of the organic to water phase ratio, MF manufacturing achieves and enhanced PFD EE and LC, compared to bulk nanoprecipitation. The number of PFD molecules per particle is similar due to a higher number of smaller-sized particles rendered by the MF technique. Particle recovery at the different parameters used is equivalent for both nanoprecipitation techniques.


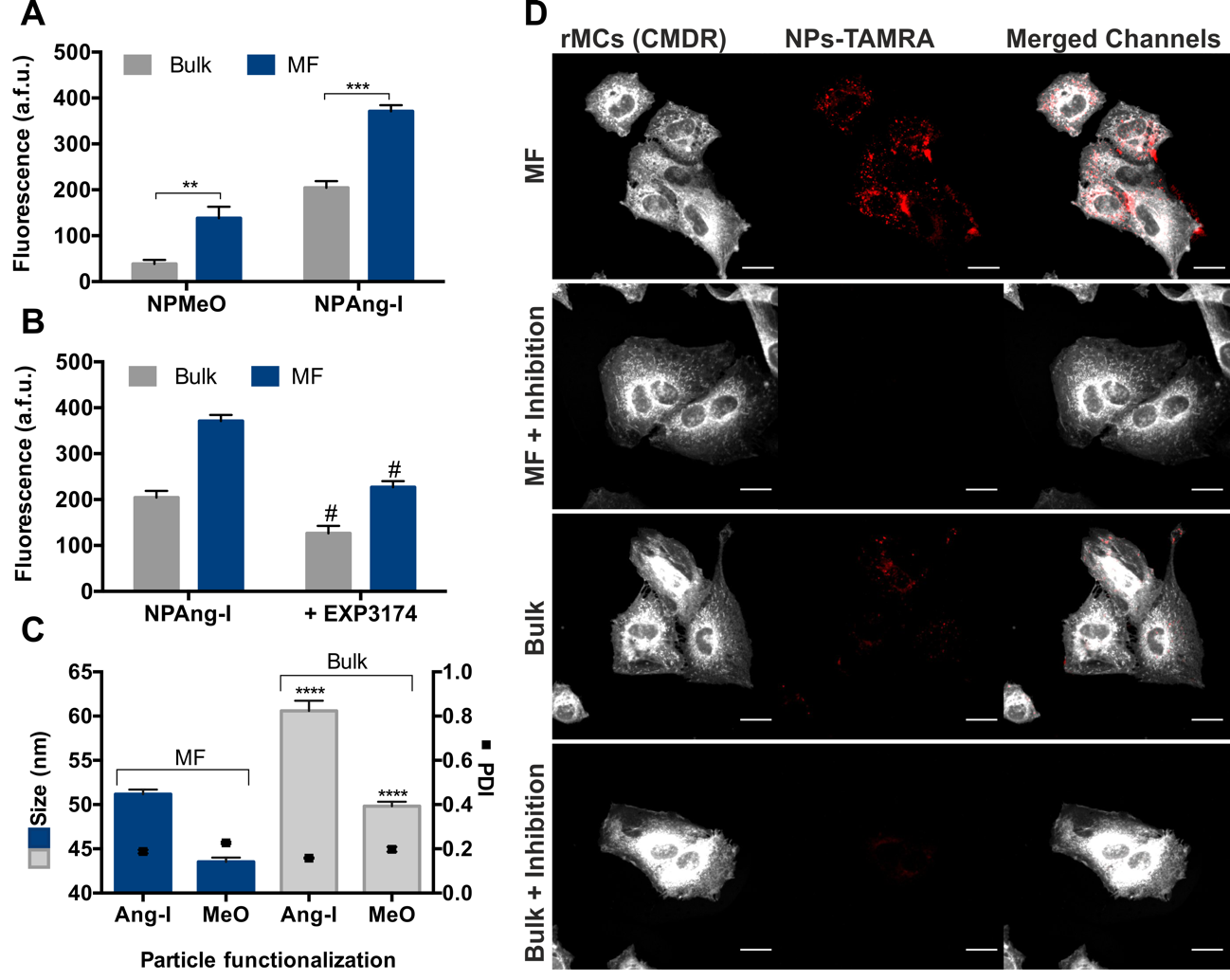


**Figure S5.** Cellular interaction of NPs prepared through microfluidic or bulk nanoprecipitation. (A) Uptake of Angiotensin-I-targeted (NPAng-I) (1) and methoxy-terminated non-targeted (NPMeO) particles in target rat mesangial cells (rMCs) analyzed through flow cytometry. (B) Specificity of the uptake shown by its suppression by EXP3174. (C) Particle size. (D) Cellular interaction of NPAng-I analyzed through CLSM. Scale bar 20 μm. Cells stained with a cell membrane stain (CMDR) and are displayed in white. NPs were prepared using a TAMRA-labelled fluorescent PLGA (1, 2) and are displayed in red. Levels of statistical significance are ****p ≤ 0.0001, ***p ≤ 0.001 and **p ≤ 0.01, or #p ≤ 0.01 comparing cells treated with NPs or NPs and EXP3174. Results are shown as mean ± SD of at least n = 3 measurements. A Student´s t-test was used to assess statistical significance. The enhanced cellular uptake of MF-manufactured NPs can be explained due to their smaller size, which falls in the ideal range for NP-cell interplay (3) (50 nm). More so, due to rapid MF mixing, the number of hydrophilic-end groups that get buried due to polymer adsorption on the particle surface is reduced (4), which can enhanced ligand-mediated targeting.


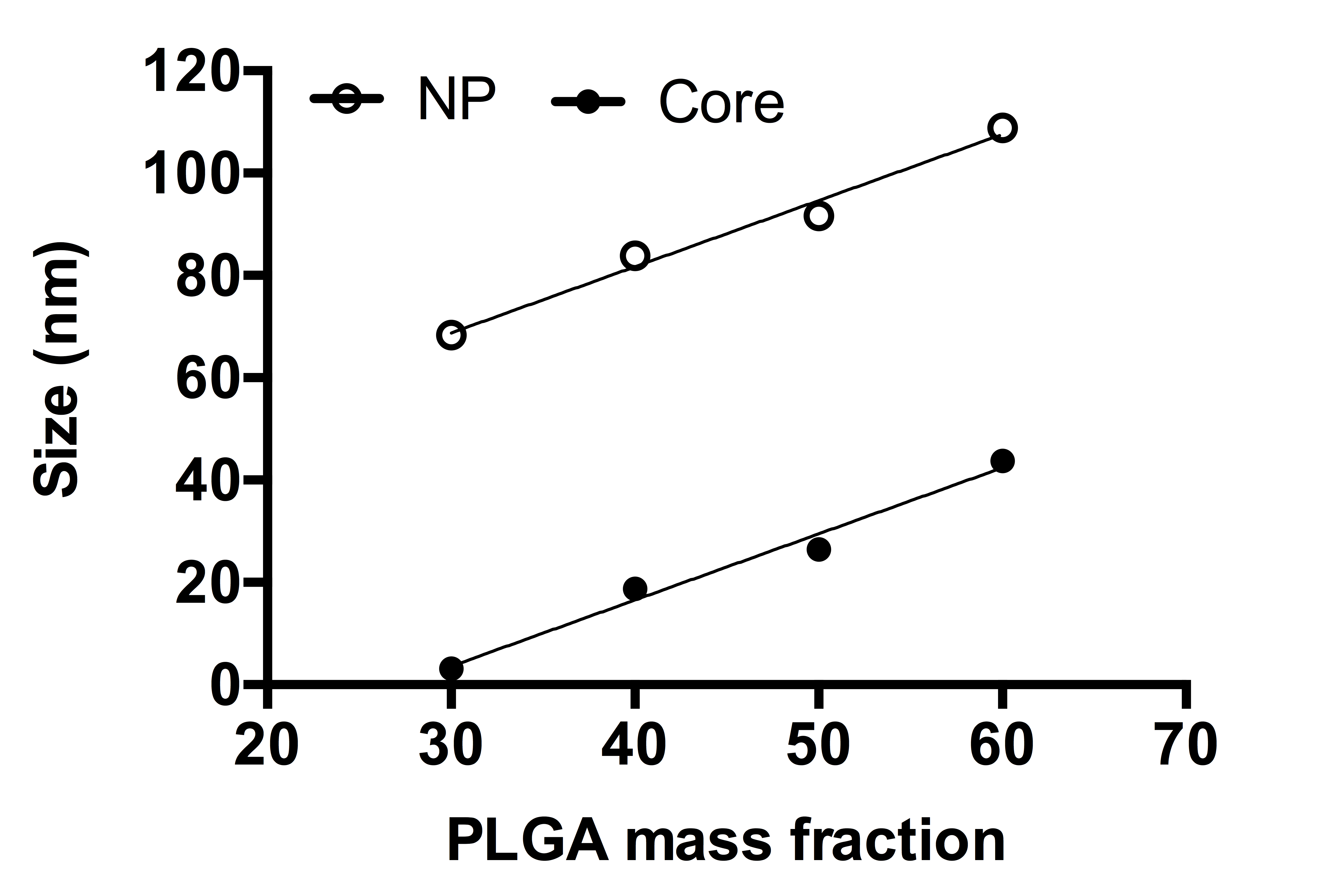


**Figure S6.** Size of NPs and their core in dependence with the PLGA mass fraction.

**
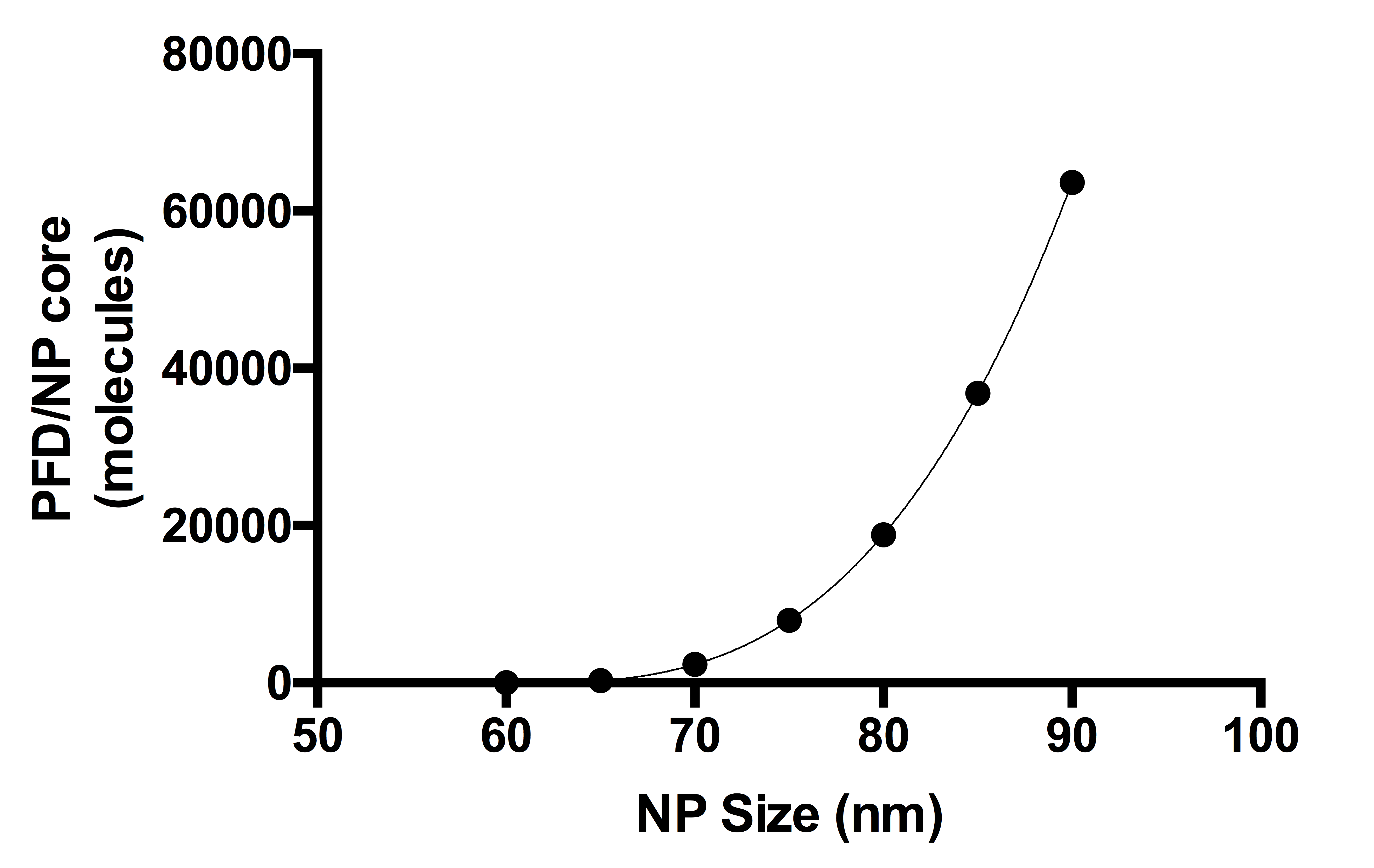
**

**Figure S7**. Theoretical number of PFD molecules per NP core calculated in dependence with the particle size. Data are fitted with a third order polynomial equation.

**
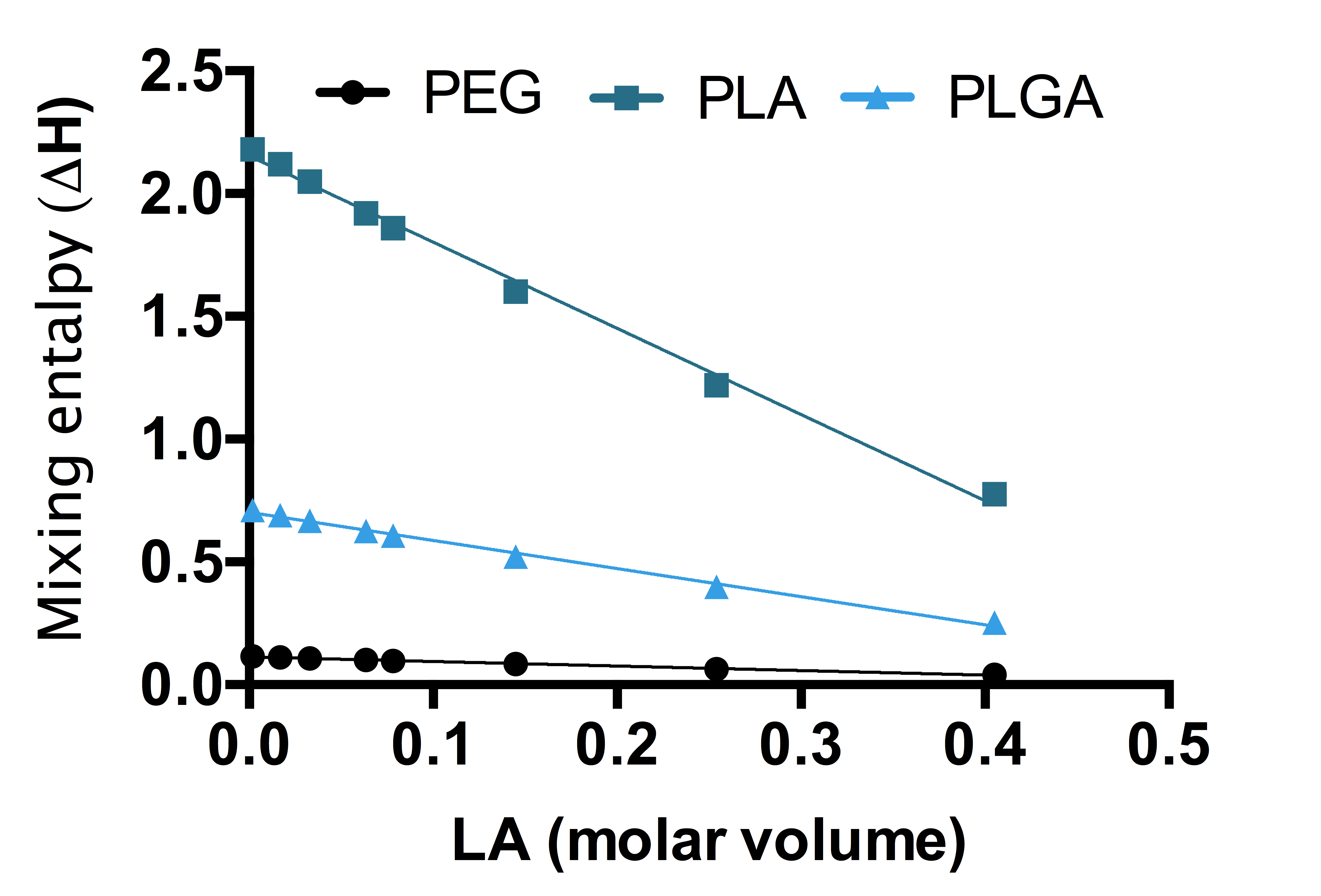
**

**Figure S8**. Mixing enthalpy of PFD and the particle forming polymers with increasing volume fractions of LA. A higher volume fraction of co-encapsulating drug, LA, decreases the mixing enthalpy of PFD with the particle-forming polymers. Data are fitted with a linear equation.


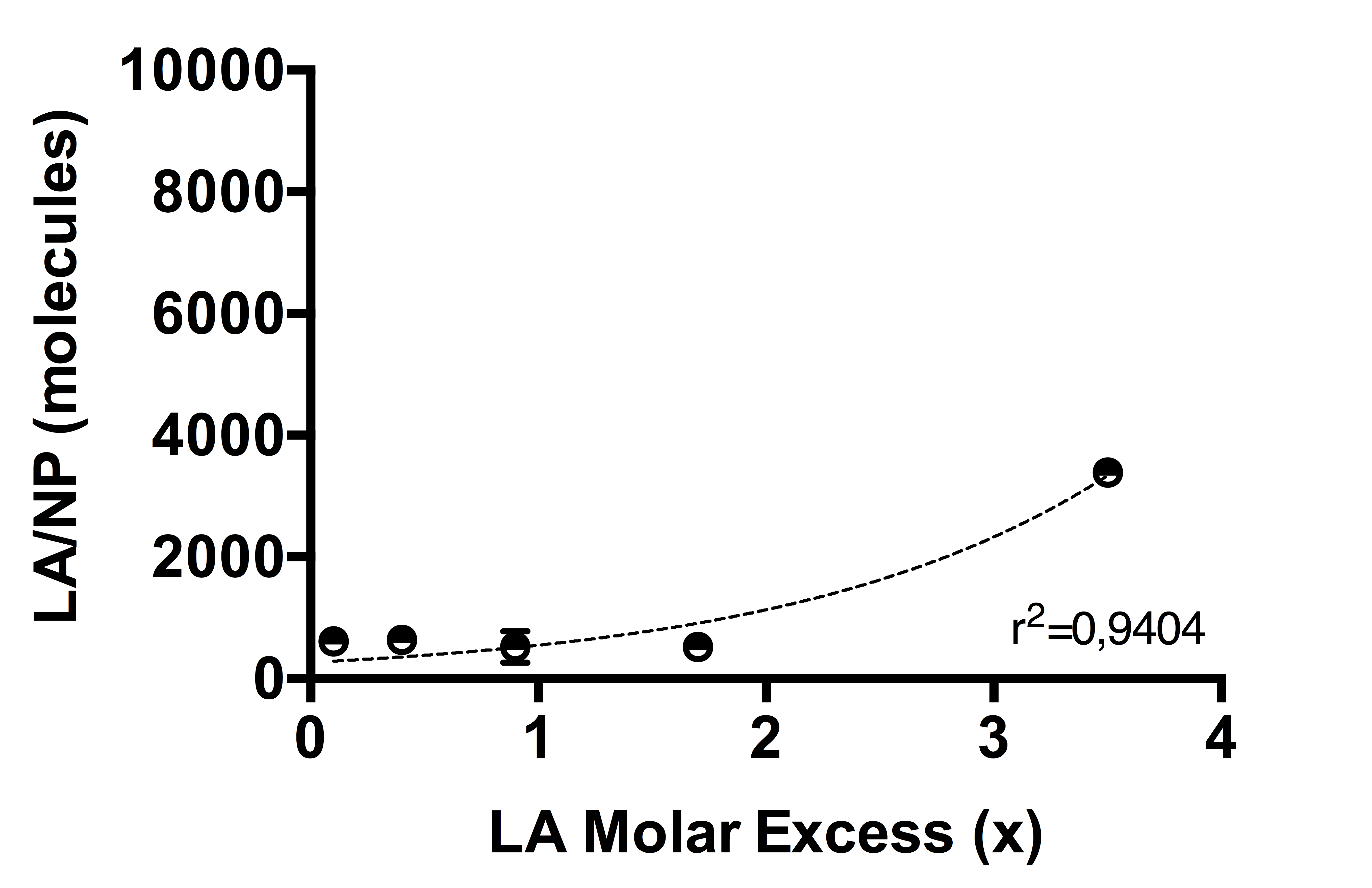


**Figure S9**. Number of LA molecules encapsulated per NP in dependence with the initially added LA.

**Table SI**. Comparison of the different miscibility prediction parameters

| Parameters | Miscibility of PFD and Polymers | Miscibility of LA and Polymers |
| --- | --- | --- |
| δ | PEG > PLGA > PLA | PEG > PLGA > PLA |
| δ_d_ | PEG > PLA > PLGA | PEG > PLA > PLGA |
| δ_p_ | PEG > PLGA > PLA | PLA > PLGA > PEG |
| δ_h_ | PLA > PEG > PLGA | PLGA > PLA > PEG |
| ΔH_M_ | PEG > PLGA > PLA | PLA > PEG = PLGA |
| ΔH_MT_ | PEG > PLGA > PLA | PEG > PLGA > PLA |
| χ_sp_ | PEG > PLGA > PLA | PEG > PLGA > PLA |

**Table SII.** PEG conformation on the particle surface

| PEG:PLGA (Mass Ratio) | S (nm^2^) | D (nm) | RF | Conformation |
| --- | --- | --- | --- | --- |
| 70:30 | 3.78 ± 0.25 | 2.19 ± 0.07 | 5.87 | Brush |
| 60:40 | 3.48 ± 0.15 | 2.10 ± 0.05 | 5.87 | Brush |
| 50:50 | 3.65 ± 0.34 | 2.15 ± 0.10 | 5.87 | Brush |
| 60:40 | 3.72 ± 0.30 | 2.18 ± 0.09 | 5.87 | Brush |

S: surface each PEG chain occupies on the particle surface; D: distance between PEG chains; RF: Flory radius.

**SUPPLEMENTARY METHODS**

**Cell culture**

The cells used in this study were cultured at 37 ºC and 5% CO_2_. Rat mesangial cells (rMCs) were a kind gift of Prof. Dr. Armin Kurtz (Institute of Physiology, University Regensburg, Regensburg, Germany). They were cultured in RPMI 1640 medium supplemented with 10% fetal bovine serum (Biowest, Nuaillé, France) and insulin-transferrin-selenium (Life Technologies, Carlsbad, CA, USA) and 100 nM hydrocortisone. They were used as target cells in uptake experiments as their expression of the target structures was shown in previous studies of our group (1, 5)

**Preparation of NPs for cellular uptake experiments**

To evaluate the cellular interaction of NPs prepared through MF and bulk nanoprecipitation, methoxy-ended NPs (NPMeO) and targeted NPs were prepared. As target moiety we selected a targeting system that we previously developed for highly specific cell recognition (1). To that end, Angiotensin-I-functionalized particles were prepared (NPAng-I) for which Lysine-terminated Angiotensin-I (Lys-Ang-I) (Genscript, Piscataway, NJ, USA) was coupled to a carboxylic-acid terminated PEG-PLA (COOH-PEG_5k_-PLA_10k_) using Ethyl-3-(3-dimethylaminopropyl)carbodiimide (EDC)/N-Hydroxysuccinimide (NHS) chemistry, as previously described (1). In short, the polymer was activated with a 25-fold molar excess of EDC/NHS for 2 hours in N,N-Dimethylformamide (DMF) prior to addition of 60-fold excess 2-mercaptoethanol for 20 minutes. Afterwards, a 1.2-fold and 5-fold molar excess of Lys-Ang-I in DMF and Diisopropylethylamine (DIPEA), respectively, were added to the activated polymer and left to react over 48 hours. The Lys-Ang-I-modified polymer (Ang-I-PEG_5k_-PLA_10k_) was purified via dialysis using a 6-8 kDa molecular weight cut-off RC dialysis membrane over 24 hours.

Targeted particles were prepared in the same manner as non-functionalized NPs, as described in the methods section of the main text using bulk or MF manufacturing. A 70:30 mass ratio of PEG-PLA to PLGA was used with 20% of the PEG-PLA being Ang-I-PEG_5k_-PLA_10k_. To ensure particle detection, a covalently fluorescently labelled PLGA (TAMRA-PLGA and CF647-PLGA for microscopy and flow cytometry experiments, respectively) was used for particle preparation, as described previously by our group (1, 2). Particle characterization was performed as described in the main text method section.

**Cellular interaction of NPs**

To assess the cellular internalization of particles prepared using different manufacturing techniques (bulk nanoprecipitation vs MF preparation) through confocal scanning laser microscopy (CLSM), rMCs were seeded at a density of 10,000 in 8-well Nunc Lab Tech^TM^ II Chamber Slide^TM^ systems (Thermo Fisher Scientific, Waltham, MA, USA). Cells were incubated for 24 hours before pre-warmed particle solutions at a concentration of 50 μg/mL in Leibovitz Medium supplemented with 0.1% bovine serum albumin (BSA) were added on top of them. After a 45-minute incubation period, the NP solution was discarded, and the cells washed with pre-warmed DBPS. The cells were stained with Cell Mask^TM^ deep red (CMDR) plasma membrane stain (Thermo Fisher Scientific, Whaltman, MA, USA) (1x) for 5 minutes at room temperature. After an additional washing step with DPBS, the cells were fixed with 4% paraformaldehyde (PFA) in DPBS for 10 minutes. Cells were washed with DPBS and imaged, using a Zeiss Axiovert 200 microscope with an LSM 510 laser-scanning device. A 63x Plan- Apochromat (NA 1.4) objective was used. To excite the NP and cell fluorescence 543 nm and 633 nm He-Ne lasers were used, respectively. A 560-615 bandpass and a 650 longpass filter were used to record the NP- and cell-associated fluorescence. To assess uptake specificity, cells were preincubated for 30 minutes with 1 mM EXP3174 (Santa Cruz Biotechnology, Dallas, TX, USA), as previously described (1), prior to particle addition.

For flow cytometry analysis cells were seeded in 30,000 cells/well in 24-well plates (Corning, Corning, NY, USA) and incubated for 48 h. Afterwards, NP solutions at a concentration of 50 μg/mL in LM supplemented with 0.1% BSA were added to the cells for 45 minutes. After the incubation, the NPs were discarded, and the cells washed with pre-warmed DPBS. Next, they were trypsinized and washed twice through centrifugation (200 *g*, 5 minutes). The cells were resuspended in ice-cold DPBS and analyzed using a CyFlow Space flow cytometer (Sysmex Partec GmbH, Goerlitz, Germany) and FloMax software. The particle-associated fluorescence was excited using a 638 nm red diode laser and recorded with a 675/30 bandpass filter. Data analysis was performed using Flowing software (Turku Centre for Biotechnology, Finland) by gating the population of viable cells and evaluating the geometrical mean fluorescence. Graph PadPrism 6.0 (GraphPad Software Inc., CA, USA) was used to assess statistical significance.

**SUPPLEMENTARY REFERENCES**

1. Maslanka Figueroa S, Veser A, Abstiens K, Fleischmann D, Beck S, Goepferich A (2019) Influenza A virus mimetic nanoparticles trigger selective cell uptake. Proc Natl Acad Sci 116:9831–9836

2. Abstiens K, Maslanka Figueroa S, Gregoritza M, Goepferich AM (2019) Interaction of functionalized nanoparticles with serum proteins and its impact on colloidal stability and cargo leaching. Soft Matter 15:709–720

3. Jiang W, Kim BYS, Rutka JT, Chan WCW (2008) Nanoparticle-mediated cellular response is size-dependent. Nat Nanotechnol 3:145–150

4. Karnik R, Gu F, Basto P, Cannizzaro C, Dean L, Kyei-Manu W, Langer R, Farokhzad OC (2008) Microfluidic Platform for Controlled Synthesis of Polymeric Nanoparticles. Nano Lett. https://doi.org/10.1021/nl801736q

5. Hennig R, Pollinger K, Tessmar J, Goepferich A (2015) Multivalent targeting of AT _1_ receptors with angiotensin II-functionalized nanoparticles. J Drug Target 23:681–689
